# Supplementary material for: Design, Synthesis and Characterization of [G10a]-Temporin SHa Dendrimers as Dual Inhibitors of Cancer and Pathogenic Microbes
Source: Biomolecules. 2022 May 31;12(6):770. doi: 10.3390/biom12060770 (PMC9221442; doi:10.3390/biom12060770)
Supplement: Supplementary file 1 [file biomolecules-12-00770-s001.zip › biomolecules-1721449-supplementary.pdf]

Design, Synthesis and Characterization of [G10a]-Temporin SHa Dendrimers as Dual Inhibitors of Cancer and Pathogenic Microbes

Arif Iftikhar Khan [1,2], Shahzad Nazir [1,2], Aaqib Ullah [1,2], Muhammad Nadeem ul Haque [1,2], Rukesh Maharjan [2], Shabana U. Simjee [2,3], Hamza Olleik [4], Elise Courvoisier-Dezord [4], Marc Maresca [4,*] and Farzana Shaheen [1,2,*]

[1] Third World Center for Science and Technology, International Center for Chemical and Biological Sciences, University of Karachi, Karachi 75270, Pakistan;

[2] H. E. J. Research Institute of Chemistry, International Center for Chemical and Biological Sciences, University of Karachi, Karachi 75270, Pakistan; khanformanite@gmail.com (A.I.K.); nazirshahzad39@gmail.com (S.N.); aqibafridi209@gmail.com (A.U.); nadeem.and.chem@gmail.com (M.N.u.H.); rukeshmaharjan2013@gmail.com (R.M.);

[3] Dr. Panjwani Center for Molecular Medicine and Drug Research, International Center for Chemical and Biological Sciences, University of Karachi, Karachi 75270, Pakistan. shabana.simjee@iccs.edu (S.U.S.)

[4] Aix-Marseille Univ, CNRS, Centrale Marseille, iSm2, 13013 Marseille, France; hamza.olleik@live.com (H.O.); elise.courvoisier-dezord@univ-amu.fr (E.C.-D.)

* Correspondence: m.maresca@univ-amu.fr (M.M.); afnan.iccs@gmail.com (F.S.);
Tel.: +33-0413945609 (M.M.); +92-3313859073 (F.S.)

Table of Contents

Figure No.	Content	Page No.
S1	UPLC Profile of Temporin- SHa peptide, [G10a]-SHa Peptide and its Dendrimers three in ACN:H_2O:TFA::60:40:0.08 at 215 nm	3-4
S2	Composition of Temporin SHa Peptide and its analogs	4
S3	Structure of Temporin-SHa Peptide	5
S4	Synthetic scheme of Temporin-SHa Peptide	6
S5	MALDI spectrum of Temporin-SHa	7
S6	^{1}H-NMR of Temporin-SHa	8
S7	NMR Data of Temporin-SHa in d6 DMSO	9-10
S8	Structure of [G10a]-SHa Peptide	11
S9	Synthetic scheme of [G10a]SHa	12
S10	ESIMS (Low Resolution Positive Mode) of [G10a]SHa	13
S11	^{1}H-NMR of [G10a]-SHa Peptide	14
S12	NMR Data of [G10a] SHa in d6 DMSO	14-17
S13	Structure of $[G10a]_2$-SHa Dendrimer	18
S14	Synthetic scheme of $[G10a]_2$SHa	19
S15	HPLC profile of $[G10a]_2$-SHa	20
S16	ESIMS (Low Resolution Positive Mode) of $[G10a]_2$SHa	20
S17	^{1}H-NMR of $[G10a]_2$-SHa Peptide	21
S18	Structure of Jeff-$[G10a]_2$-SHa Dendrimer	22
S19	Synthetic scheme of Jeff-$[G10a]_2$SHa	23

**S20**	**HPLC profile of Jeff-[G10a]$_2$SHa**	**24**
**S21**	**ESIMS (Low Resolution Positive Mode) of Jeff-[G10a]$_2$SHa**	**25**
**S22**	**^{1}H-NMR of Jeff-[G10a]$_2$-SHa Peptide**	**26**
**S23**	**Structure of [G10a]$_3$-SHa Dendrimer**	**27**
**S24**	**Synthetic scheme of [G10a]$_3$SHa**	**28-29**
**S25**	**HPLC profile of [G10a]$_3$SHa**	**30**
**S26**	**ESIMS (Low Resolution Positive Mode) of [G10a]$_3$SHa**	**31**
**S27**	**^{1}H-NMR of [G10a]$_3$-SHa Peptide**	**32**
**S28**	**IR spectra of dendrimer peptides**	**33**
**S29**	**UV-Visible spectra of dendrimer peptides**	**34**

**Peptide Dendrimers**	**UPLC Profile of Temporin-SHa and its analogs in ACN:H$_2$O:TFA::60:40:0.08 at 215 nm**
**1**	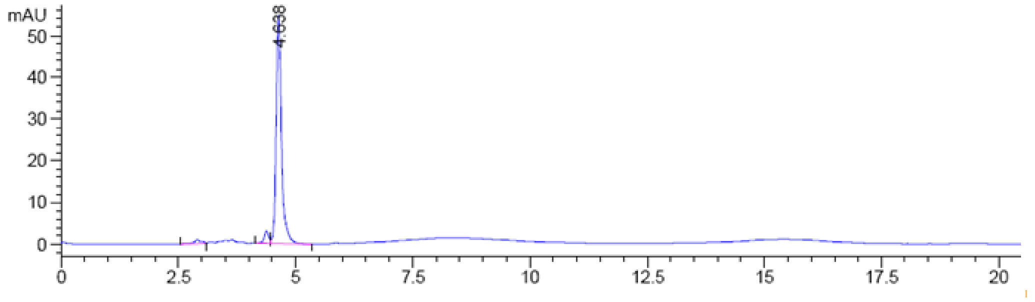 UPLC Profile of Temporin-SHa**.** C4 reversed-phase analytical column, ACN/H_2O/TFA 60 % /40 % / 0.08 %
**2**	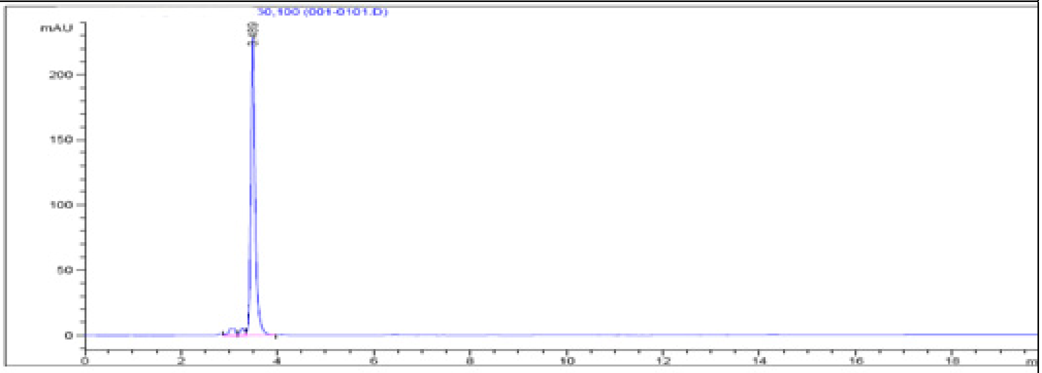 UPLC Profile of [G10a] SHa**.** C4 reversed-phase analytical column, ACN/H_2O/TFA 60 % /40 % / 0.082

3	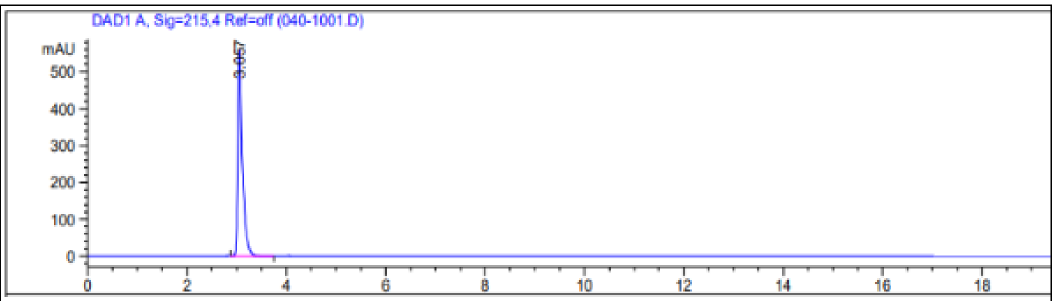<br>UPLC Profile of JEFF-[G10a] SHa. C4 reversed-phase analytical column, ACN/H_2O/TFA 60 % /40 % 0.082 %
4	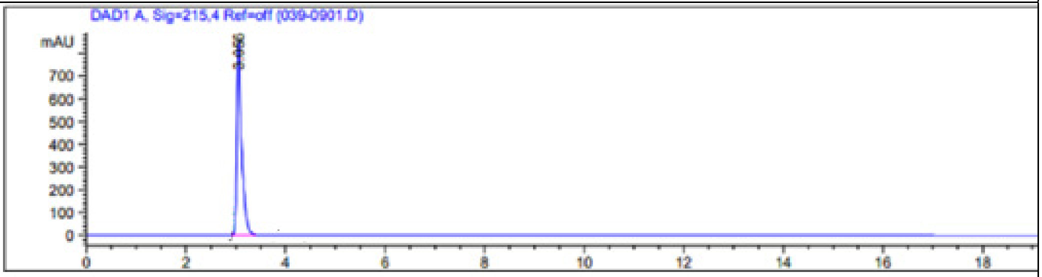<br>UPLC Profile of JEFF-$[G10a]_3$ SHa. C4 reversed-phase analytical column, ACN/H_2O/TFA 60 % /40 % 0.082 %
5	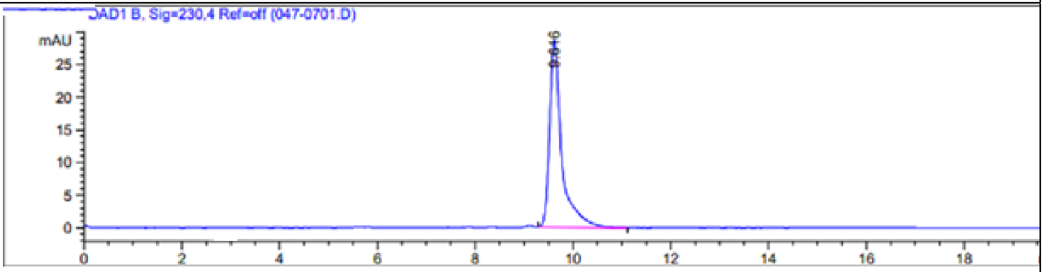<br>UPLC Profile of $[G10a]_2$ SHa. C4 reversed-phase analytical column, ACN/H_2O/TFA 60 % /40 % / 0.0
**Figure S1: UPLC Profile of Peptide Dendrimers 1-4 in ACN:H_2O:TFA::60:40:0.08 at 215 nm**	

SEQ NO	AA_1	AA_2
**SEQ ID NO: 1**	**$R^4 = R^7 = R^{10}$ Gly,**	**-**
**SEQ ID NO: 2**	**$R^4 = R^7 =$ Gly,**	**R^{10} = D-Ala**
**SEQ ID NO: 3**	**$R^4 = R^7 = R^{17} = R^{20}$ Gly,**	**$R^{10} = R^{23}$ = D-Ala**
**SEQ ID NO: 4**	**JEF-$R^4 = R^7 = R^{17} = R^{20}$ Gly,**	**$R^{10} = R^{23}$ = D-Ala**
**SEQ ID NO: 5**	**$R^4 = R^7 = R^{17} = R^{20}$ $R^{30} = R^{33} =$ Gly,**	**$R^{10} = R^{23} = R^{36}$ = D-Ala**

Figure-S2: Composition of Temporin-SHa Peptide and its analogs

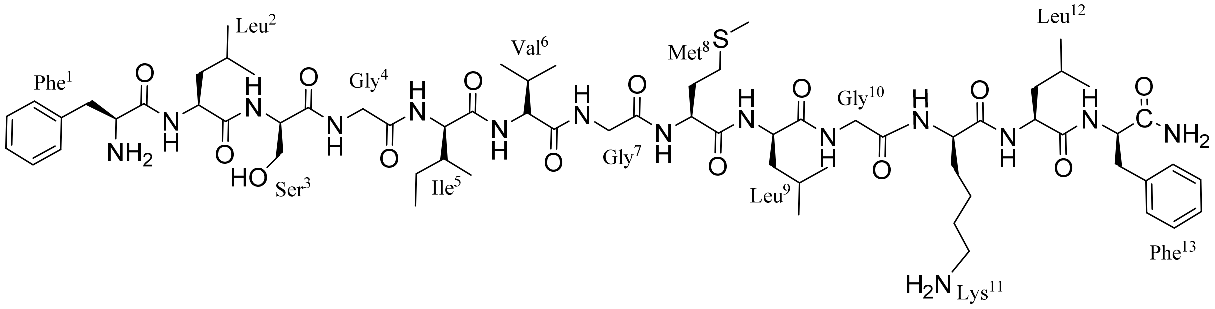

FigureS3: Structure of Temporin-SHa.

Temporin SHa

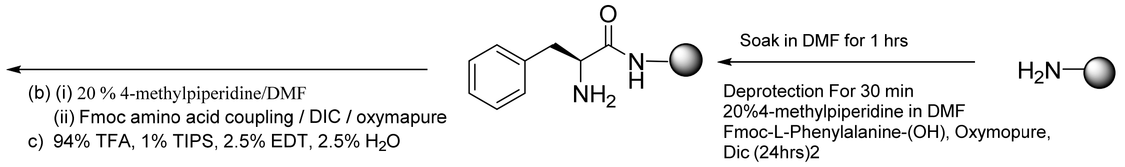

2. Fmoc-L-Leucine + Oxymapure+DIC,3hr
   20% 4-mehylpipridine, 30minuts

3. Fmoc-L-Lysine+ Oxymapure+DIC3hr
   20% 4-methylpipridine, 30minuts

4. Fmoc-Gly+ Oxymapure+DIC,3hr
   20% 4-methylpipridine, 30minuts

5. Fmoc-L-Leucine+ Oxymapure+DIC,3hr
   20% 4-methylpipridine, 30minuts

6. Fmoc-L-Methionine+ Oxymapure+DIC,3hr
   20% 4-methylpipridine, 30minuts

7. Fmoc-Glycine+ Oxymapure+DIC,3hr
   20% 4-methylpipridine, 30minuts

8. Fmoc-L-Valine+ Oxymapure+DIC,3hr
   20% 4-methylpipridine, 30minuts

9. Fmoc-L-Isoleucine+ Oxymapure+DIC,3hr
   20% 4-methylpipridine, 30minuts

10. Fmoc-Gly+ Oxymapure+DIC,3hr
   20% 4-methylpipridine, 30minuts

11. Fmoc-L-Serine+ Oxymapure+DIC,3hr
   20% 4-methylpipridine, 30minuts

12. Fmoc-L-Leucine+ Oxymapure+DIC,3hr
   20% 4-methylpipridine, 30minuts

13. Fmoc-L-Phenylalanine+ Oxymapure+DIC,3hr
   20% 4-methylpipridine, 30minuts

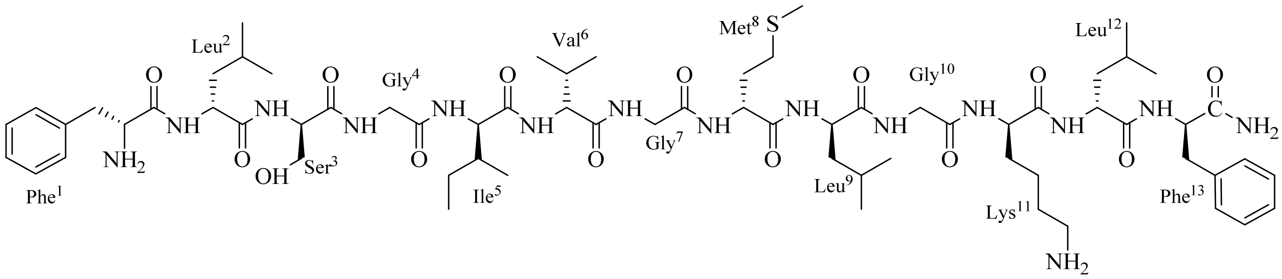

Chemical Formula: $C_{67}H_{109}N_{15}O_{14}S$
Exact Mass: 1379.7999
Molecular Weight: 1380.7600

Figure S4. Synthetic scheme of Temporin-SHa

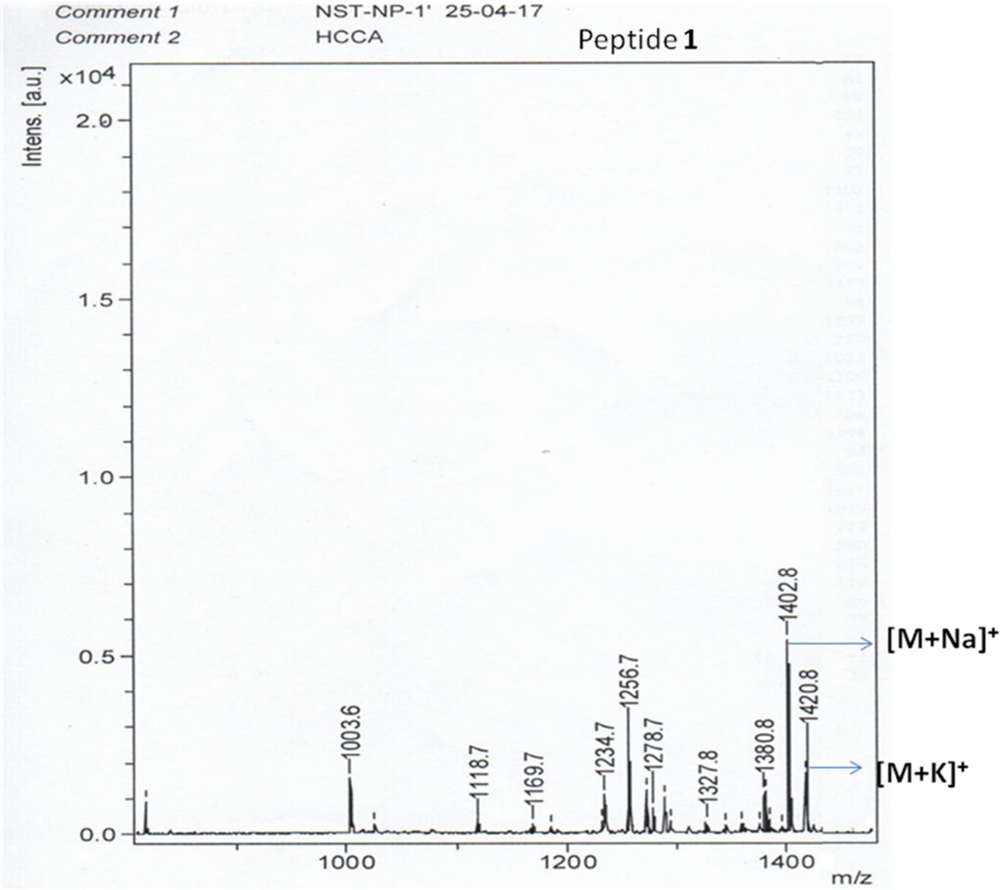

Figure S5. MALDI mass spectra of Temporin-SHa

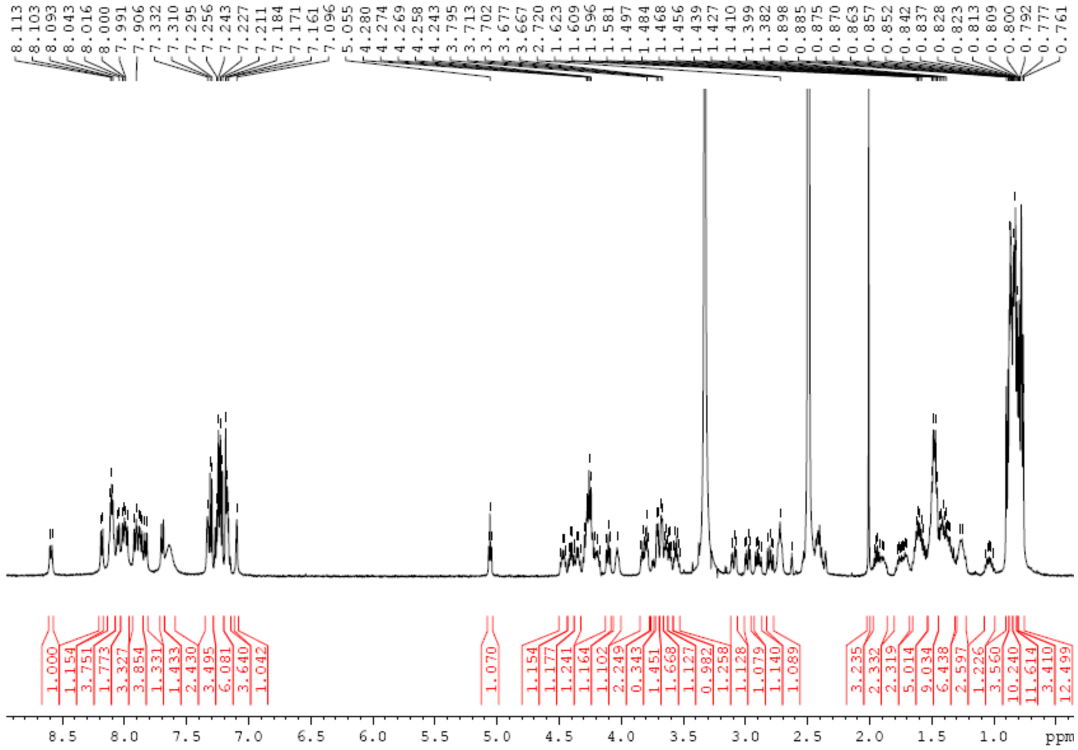

Figure S6. NMR Spectra of Temporin-SHa

Residue	Position	Temporin-SHa ^{1}H-NMR	 *J* (Hz)	 ^{13}C-NMR
Phe[1]		4.04 s		53.4
		2.88 dd, 3.09 dd	8.2, 14.2 & 4.38, 14.1	37.1
				134.9
	2-6	(7.17-7.18)m		126.2-129.5
	CO			167.7
	NH_2	overlapped		
*Leu[2]		4.46 dd	8.5, 14.0	51.0
		(1.35-1.49) m		41.0
		(1.62-1.38) m		24.1
	δ_1 CH_3	(0.75-0.80) m		21.5
	δ_2 CH_3	(0.80-0.90) m		23.1
	CO			169.
	NH	8.61 d	8.0	
Ser[3]	CH	4.27 m		55.2
	CH2	3.61p, 3.55p	5.5, 5.6	61.8
	CO			171.18
	NH	8.18 d	7.4	
	OH	5.06t	5.5	
*Gly[4]		3.82m, 3.67m		42.0-41.8
	CO			171.2
	NH	8.11 m		
Ile[5]		4.24 m		56.7
		1.48* m		26.7
		1.69 m		36.5
	δ1	0.79 m		14.7
	δ2	0.76 m		10.9
	CO			168.2
	NH	7.83 d	8.5	
Val[6]		4.10 t	7.5	58.0
		1.93 m		30.3
		(0.80-0.82) m		18.4, 19.1
	CO			170.6
	NH	7.88 m		
Gly[7]		3.82m, 3.67m		42.0-41.8
	CO			
	NH	8.1 m		
Met[8]		4.37 m		52.0

		2.39 m		29.4
		(1.61-1.47) m		31.6
	δ	1.99 s		15.3
	CO			171.1
	NH	7.98 m		
*Leu[9]		4.25 m		51.3
		(1.35-1.49) m		40.3
		(1.62-1.38) m		24.1
	δ_1 CH_3	(0.75-0.80) m		21.5
	δ_2 CH_3	(0.80-0.90) m		23.1
	CO			171.1
	NH	8.09 m		
Gly[10]		3.82m, 3.67m		42.0-41.8
	CO			168.9
	NH	8.10 m		
Lys[11]		4.23 dd	8.5, 13.5	51.9
		(1.40-1.47) m		32.0
		1.24 m		22.1
		1.50 m		26.6
		2.71 m		39.0
	CO			167.6
	NH	8.06 d	7.6	
	NH_2	overlapped		bs
*Leu[12]		4.22 m		51.1
		(1.35-1.49) m		40.6
		(1.62-1.38) m		24.1
	δ_1 CH_3	(0.75-0.80) m		21.5
	δ_2 CH_3	(0.80-0.90) m		23.1
	CO			171.07
	NH	7.98 m		
Phe[13]		4.42 seq	5.5, 8.5	53.1
		2.80 dd, 2.99 dd	8.04, 14.1 & 8.7, 13.7	37.6
				137.7
	2-6	(7.22-7.25) m		126.2-129.5
	CO			170.70
	NH	7.70 d	8.1	
	NH_2	7.64	bs	

Table S7. NMR data of Temporin-SHa in DMSO-d_6 (in ppm, J in Hz)

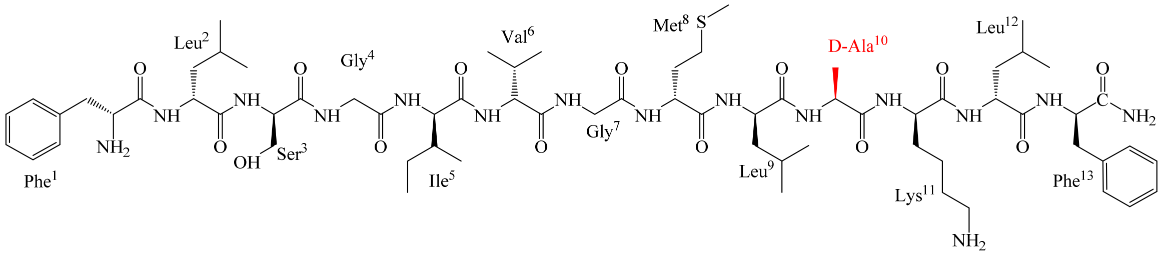

FigureS8: Structure of **[G10a]-SHa**.

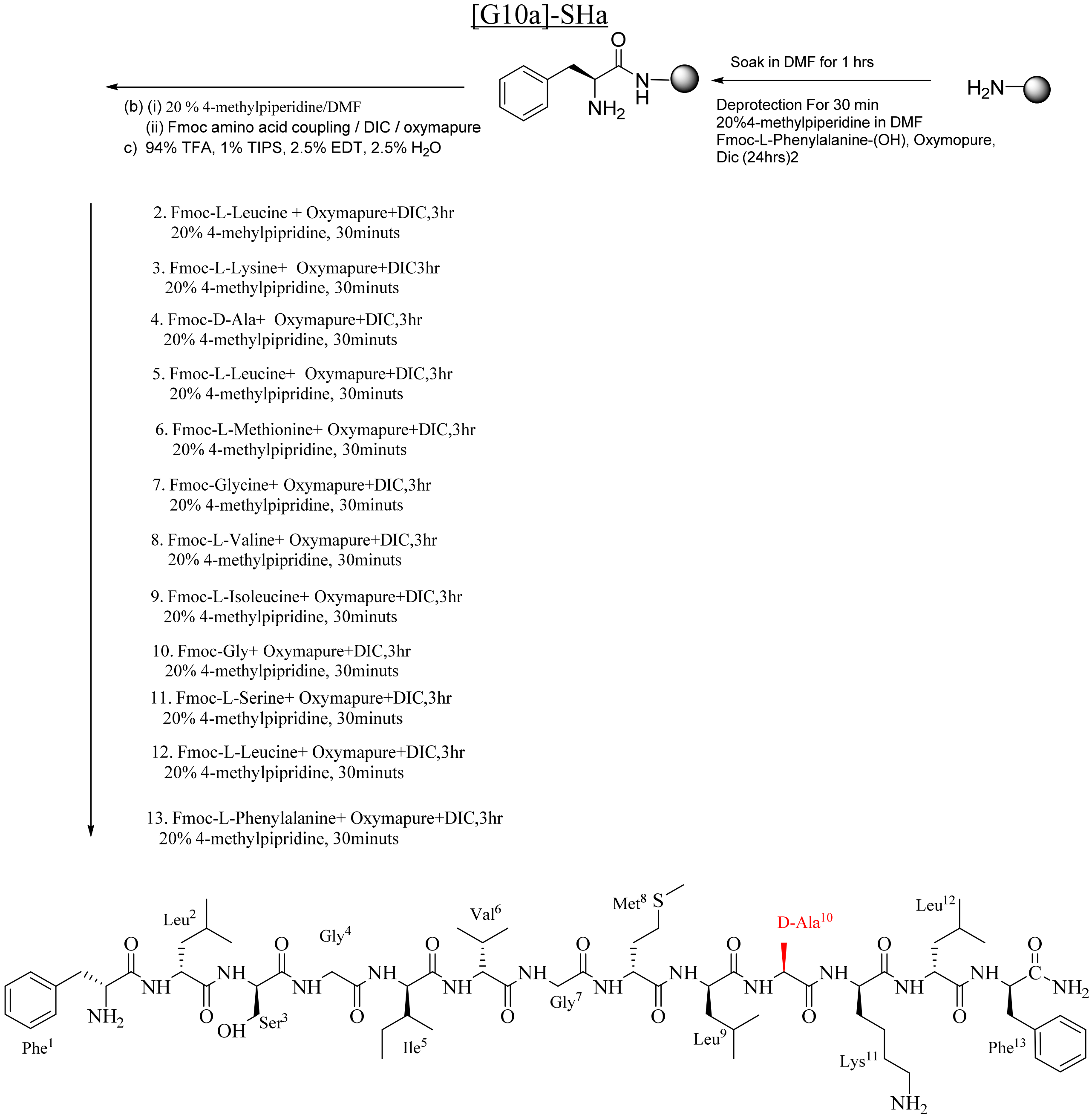

Chemical Formula: $C_{68}H_{111}N_{15}O_{14}S$

Exact Mass: 1393.8156

Molecular Weight: 1394.7870

Figure S9. Synthetic scheme of [G10a]-SHa

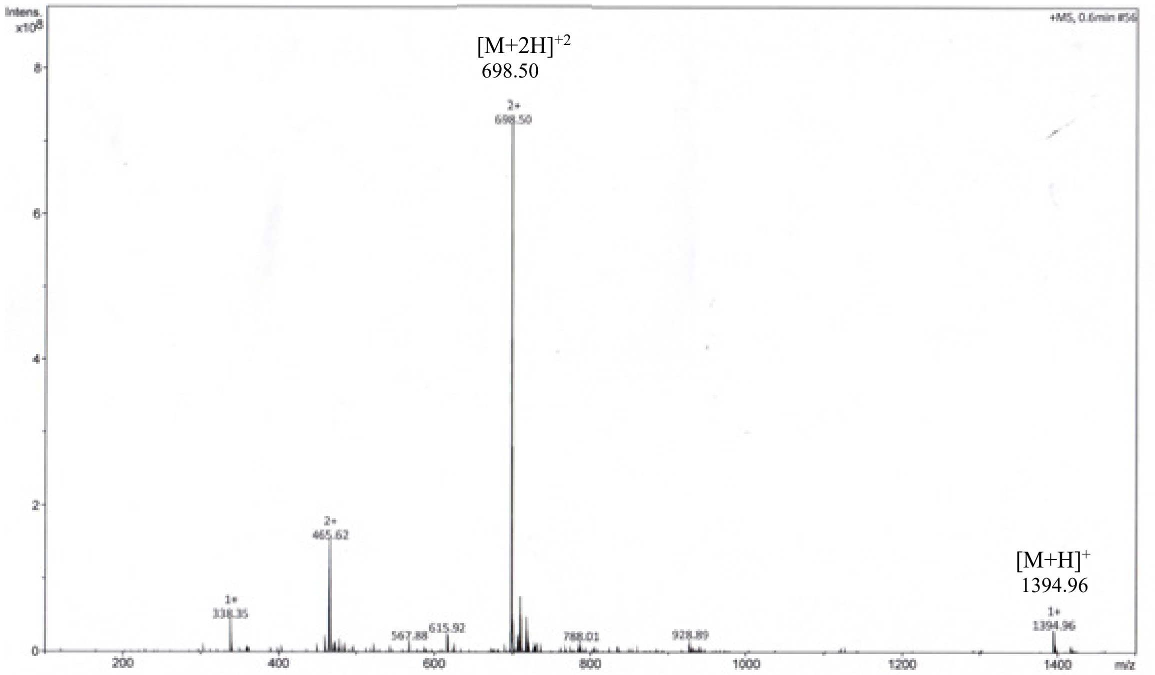

Figure S10: ESIMS (Low Resolution Positive Mode) of [G10a]-SHa

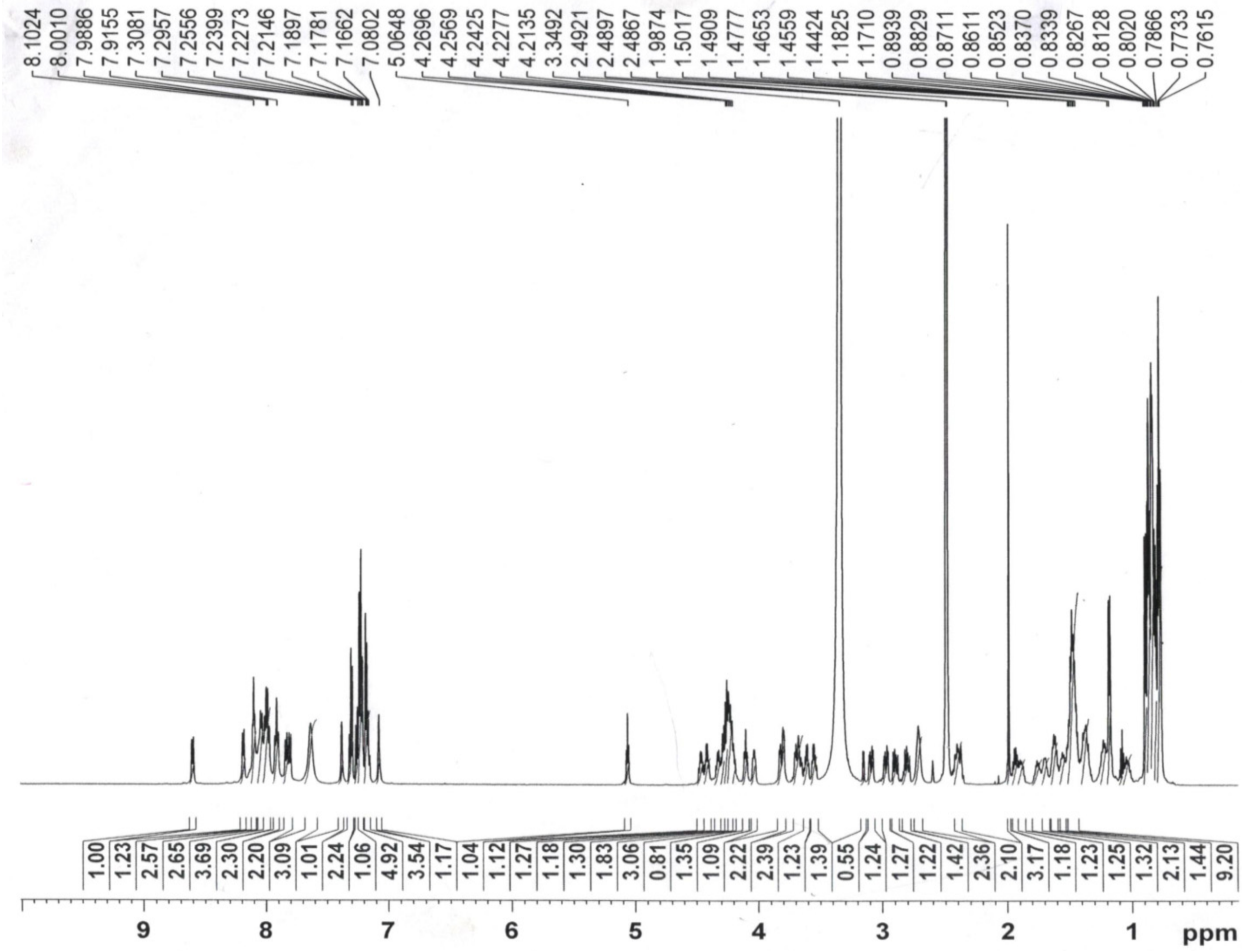

Figure S11: ^{1}H-NMR of [G10a]-SHa

Residue	Position	[G10a] SHa		
		600 MHz ^{1}H-NMR	*J* (Hz)	150 MHz ^{13}C-NMR
Phe[1]		4.04		53.20
		2.87 dd,, 3.07 dd	8.2, 14.2 & 4.38, 14.1	37.00
				134.7
	2-6	(7.17-7.18) m		129.2
	CO			167.7

	NH_2			
*Leu[2]		4.46 dd	8.22, 14.3	51.0
		(1.43-1.55) m		41.0
		(1.61-1.04) m		24.2
	δ_1 CH_3	(0.75-0.80) m		21.5,
	δ_2 CH_3	(0.80-0.90) m		23.0
	CO			169.
	NH	8.6 d	9.3	
Ser[3]	CH	4.26 m		55.1
	CH2	3.60 p, 3.54 p	5.5, 5.5	61.5
	CO			171.18
	NH	8.00 m		
	OH	5.06 t	5.4	
Gly[4]		3.82 m, 3.68 m		42.1
	CO			171.2
	NH	8.11 m		
Ile[5]		4.24 m		56.7
		1.45* m		26.6
		1.69 m		36.5
	δ1	0.79 m		14.6
	δ2	0.76 m		10.9
	CO			168.2
	NH	7.84 d	8.4	
Val[6]		4.10 t	7.5	58.0
		1.93 m		30.2
		(0.80-0.82) m		18.9, 19.2
	CO			170.6
	NH	7.90 m		

Gly[7]		3.67 m, 3.80 m		41.9
	CO			168.9
	NH	7.90 m		
Met[8]		4.32 (m)		52.0
		2.39 m		29.4
		(1.75-1.85)m		31.8
	δ	1.97 m		15.3
	CO			171.1
	NH	8.19 d	7.3	
*Leu[9]		4.21 m		51.35
		(1.43-1.55) m		40.6
		(1.61-1.04) m		24.0
	δ_1 CH_3	(0.75-0.80) m		21.6
	δ_2 CH_3	(0.80-0.90) m		22.9
	CO			171.1
	NH	8.0 m		
Ala[10]		4.25 m		48.31
		1.18 d	6.9	18.42
	CO			171.18
	NH	7.92 m		
Lys[11]		4.26m		51.1
		(1.40-1.47)m		31.30
		1.22 m		22.17
		1.50 m		26.64
		2.71 m		38.79
	CO			167.6
	NH	7.99 m		
	NH_2			bs

*Leu[12]		4.22 m		51.4
		(1.43-1.55) m		40.4
		(1.61-1.04) m		24.0
	δ1 CH_3	(0.75-0.80) m		21.5
	δ2 CH_3	(0.80-0.90) m		23.2
	CO			171.07
	NH	8.07 m		
Phe[13]		4.40 dd	8.3, 13.5	53.5
		2.78dd , 2.96dd	8.04, 14.1 & 8.7, 13.7	37.6
				137.6
	2-6	7.22-7.25m		127.1-128.0
	CO			170.70
	NH	7.81 d	8.4	
	NH_2			

Figure S12: NMR Data of [G10a] SHa in d_6 DMSO.

[G10a]$_2$-SHa

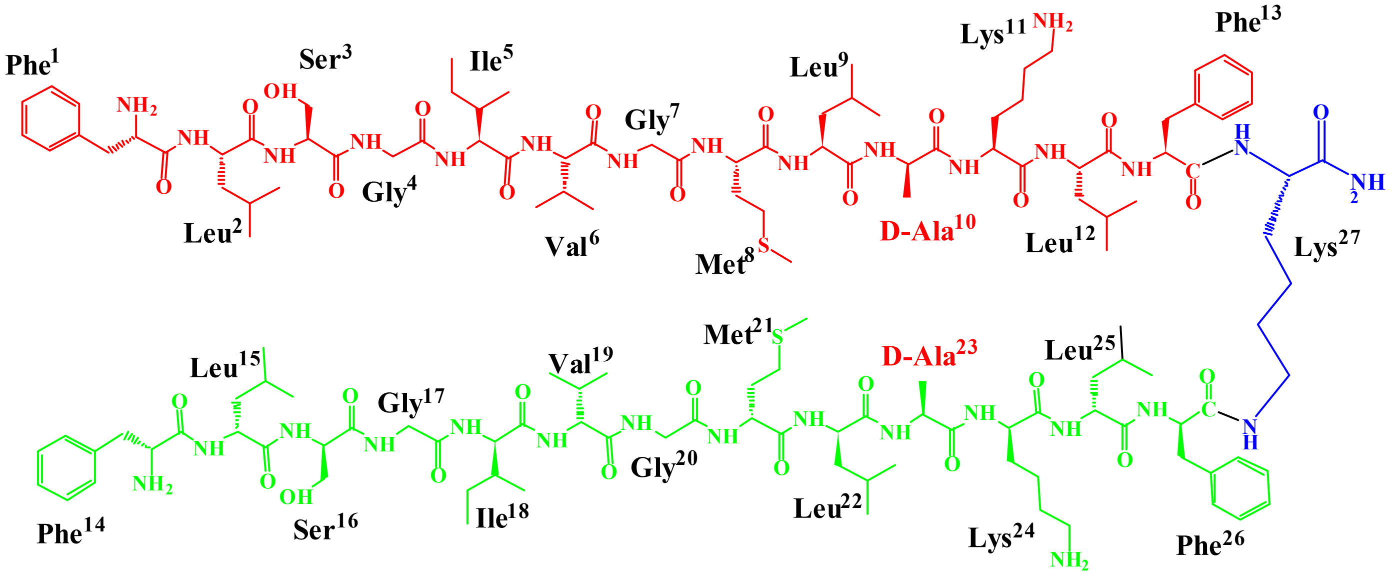

FigureS13: Structure of **[G10a]$_2$-SHa**.

[G10a]$_2$-SHa

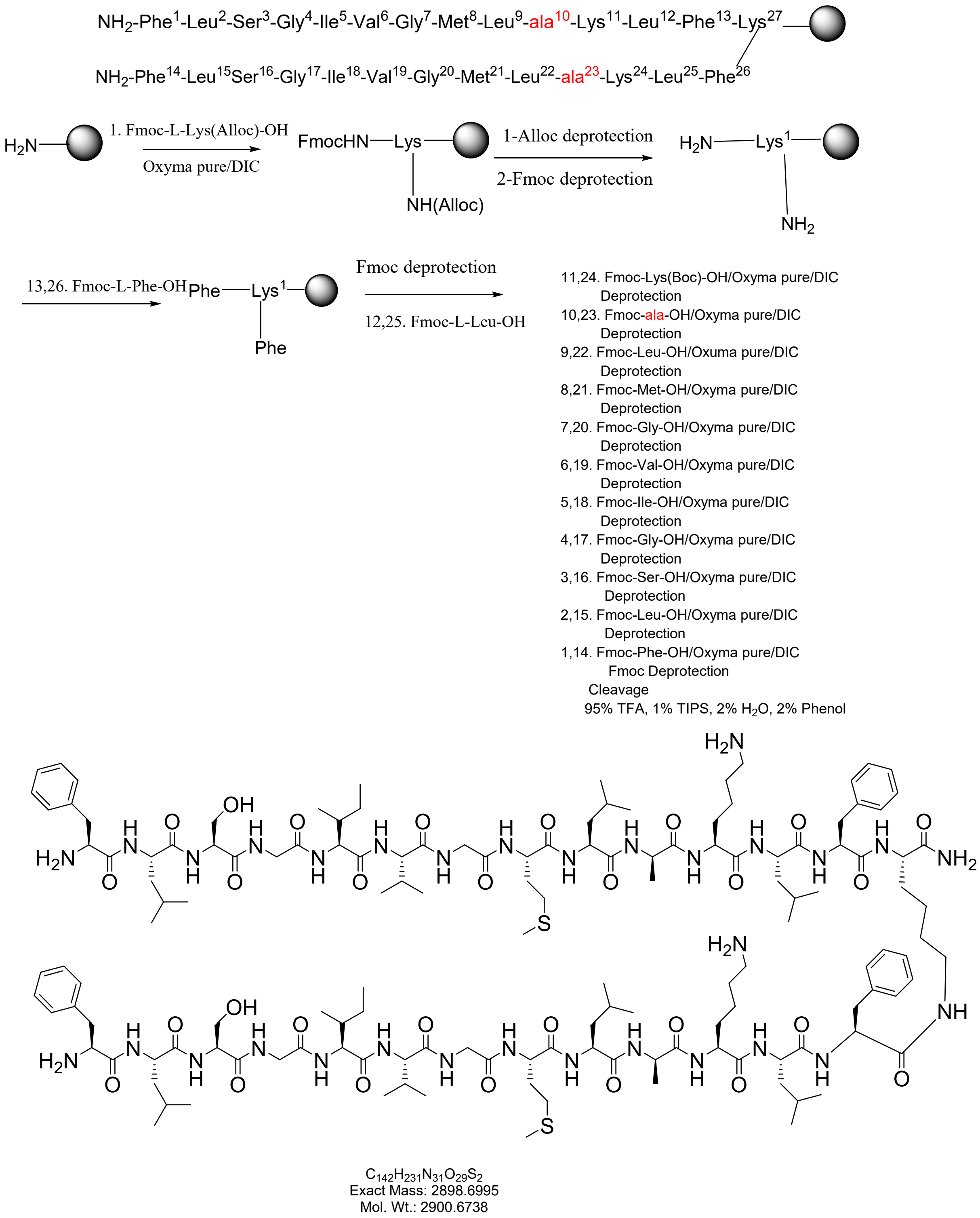

Figure S14. Synthetic scheme of [G10a]$_2$SHa

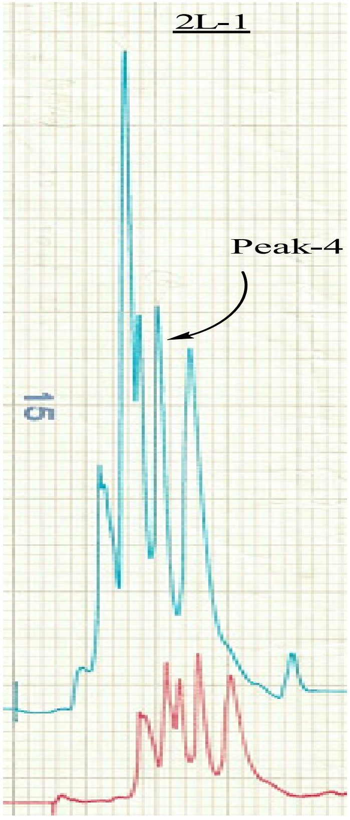

Figure S15: HPLC profile of [G10a]$_2$-SHa

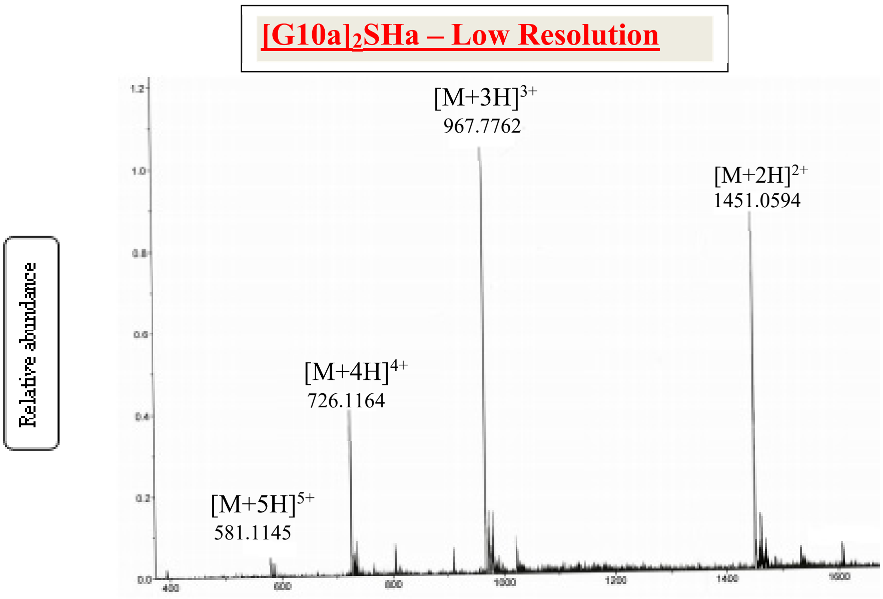

Figure S16: ESIMS (Low Resolution Positive Mode) of [G10a]$_2$SHa

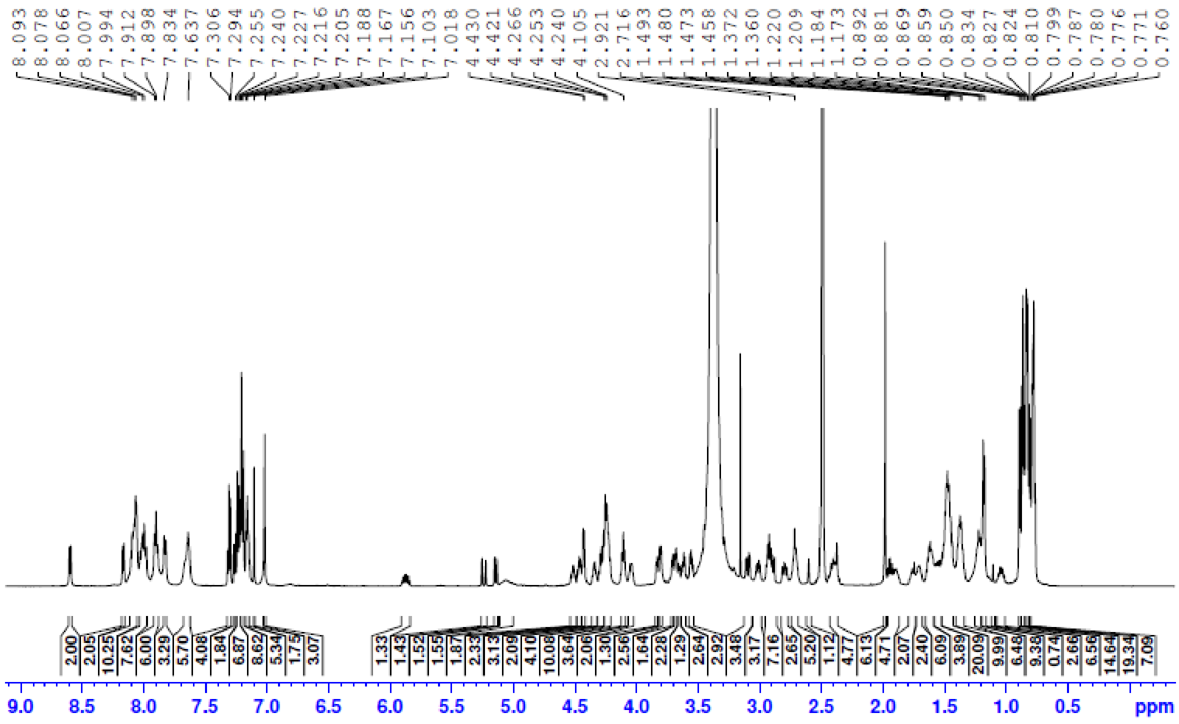

Figure S17: ^{1}H-NMR of [G10a]$_2$SHa

Jeff-[G10a]₂-SHa

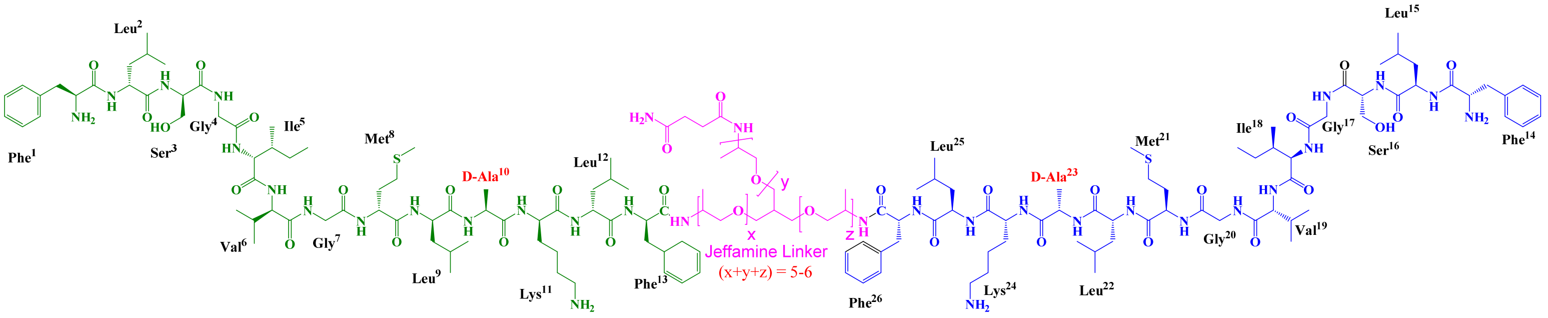

Figure S18: Structure of **Jeff-[G10a]₂-SHa**

JEFF-[G10a]$_2$-SHa

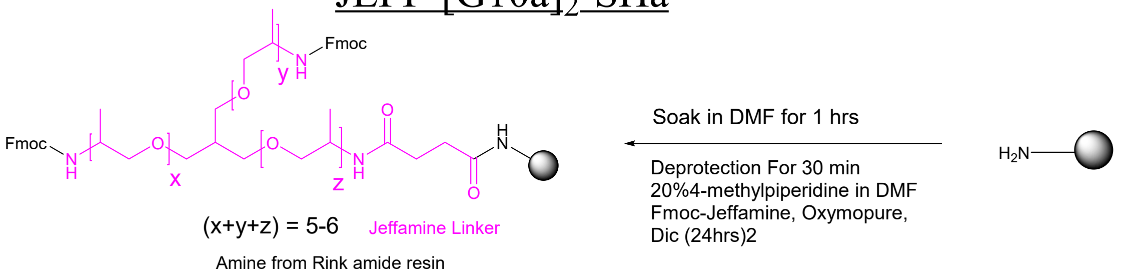

13,26. Fmoc-L-Phe + Oxymapure+DIC,3hr
20% 4-mehylpipridine, 30minuts

12,25. Fmoc-L-Leucine + Oxymapure+DIC,3hr
20% 4-mehylpipridine, 30minuts

11,24. Fmoc-L-Lysine+ Oxymapure+DIC3hr
20% 4-methylpipridine, 30minuts

10,23. Fmoc-D-Ala+ Oxymapure+DIC,3hr
20% 4-methylpipridine, 30minuts

9,22. Fmoc-L-Leucine+ Oxymapure+DIC,3hr
20% 4-methylpipridine, 30minuts

8,21. Fmoc-L-Methionine+ Oxymapure+DIC,3hr
20% 4-methylpipridine, 30minuts

7,20. Fmoc-Glycine+ Oxymapure+DIC,3hr
20% 4-methylpipridine, 30minuts

6,19. Fmoc-L-Valine+ Oxymapure+DIC,3hr
20% 4-methylpipridine, 30minuts

5,18 Fmoc-L-Isoleucine+ Oxymapure+DIC,3hr
20% 4-methylpipridine, 30minuts

4,17. Fmoc-Gly+ Oxymapure+DIC,3hr
20% 4-methylpipridine, 30minuts

3,16. Fmoc-L-Serine+ Oxymapure+DIC,3hr
20% 4-methylpipridine, 30minuts

2,15 Fmoc-L-Leucine+ Oxymapure+DIC,3hr
20% 4-methylpipridine, 30minuts

1,14. Fmoc-L-Phenylalanine+ Oxymapure+DIC,3hr
20% 4-methylpipridine, 30minuts

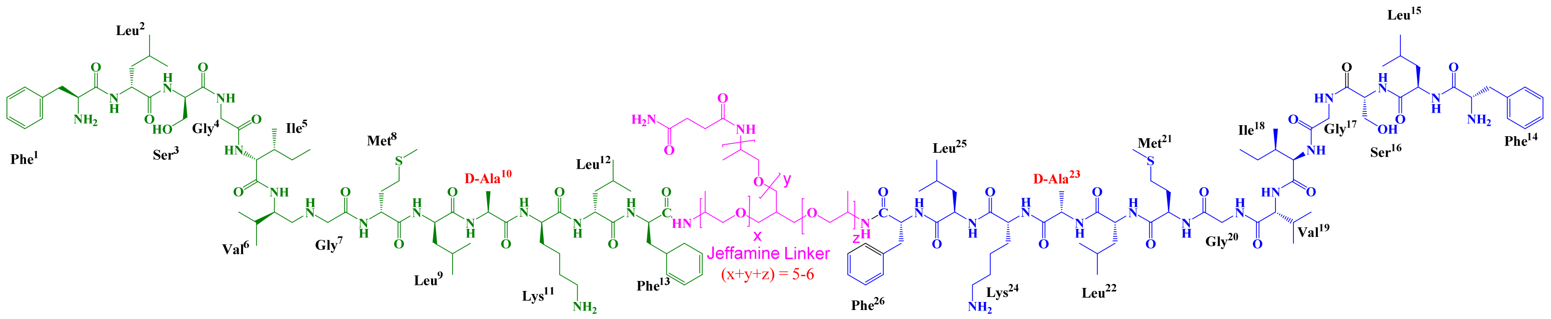

Figure S19. Synthetic scheme of Jeff-[G10a]$_2$SHa

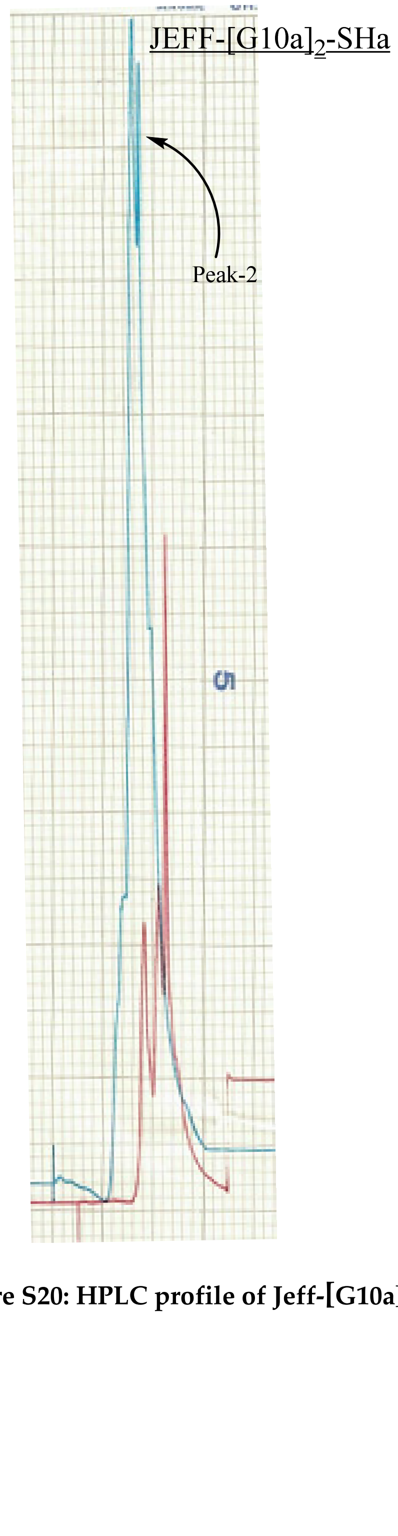

Figure S20: HPLC profile of Jeff-[G10a]$_2$SHa

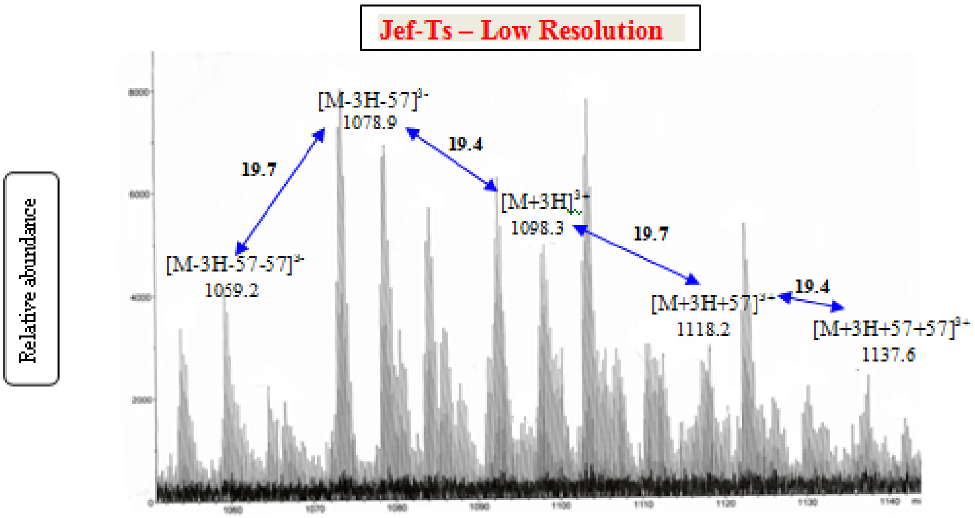

Figure S21: ESIMS (Low Resolution Positive Mode) of Jeff-[G10a]$_2$SHa

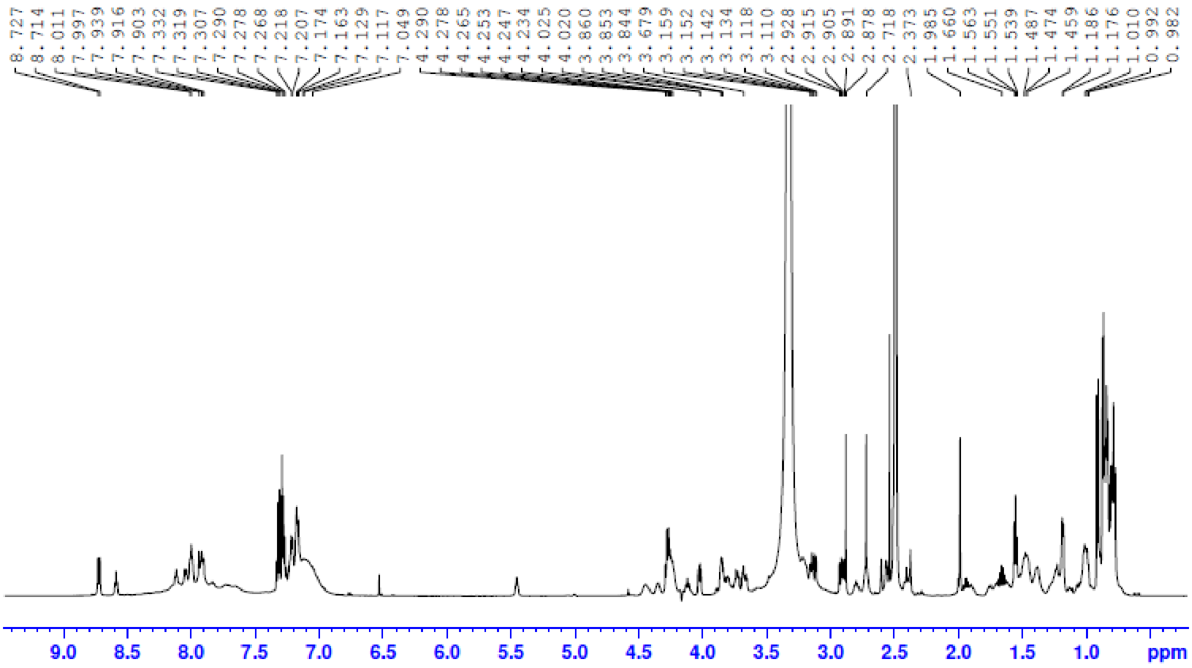

Figure S22: ^{1}H-NMR of Jeff-[G10a]$_2$SHa

[G10a]3-SHa

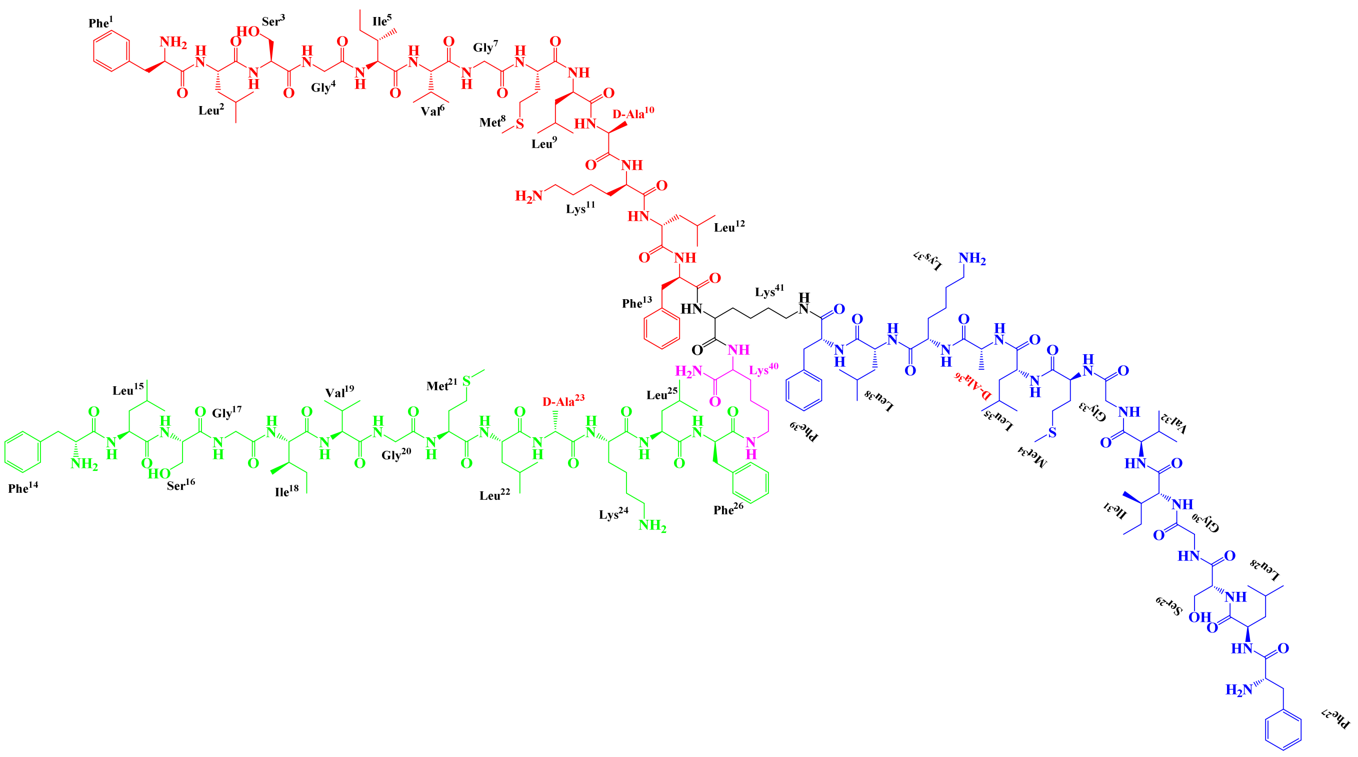

FigureS23: Structure of **[G10a]3-SHa**

[G10a]$_3$-SHa

NH$_2$-Phe^{1}-Leu^{2}-Ser^{3}-Gly^{4}-Ile^{5}-Val^{6}-Gly^{7}-Met^{8}-Leu^{9}-ala^{10}-Lys^{11}-Leu^{12}-Phe^{13}-Lys^{41}

NH$_2$-Phe^{14}-Leu^{15}-Ser^{16}-Gly^{17}-Ile^{18}-Val^{19}-Gly^{20}-Met^{21}-Leu^{22}-ala^{23}-Lys^{24}-Leu^{25}-Phe^{26}-Lys^{40}

NH$_2$-Phe^{27}-Leu^{28}-Ser^{29}-Gly^{30}-Ile^{31}-Val^{32}-Gly^{33}-Met^{34}-Leu^{35}-ala^{36}-Lys^{37}-Leu^{38}-Phe^{39}

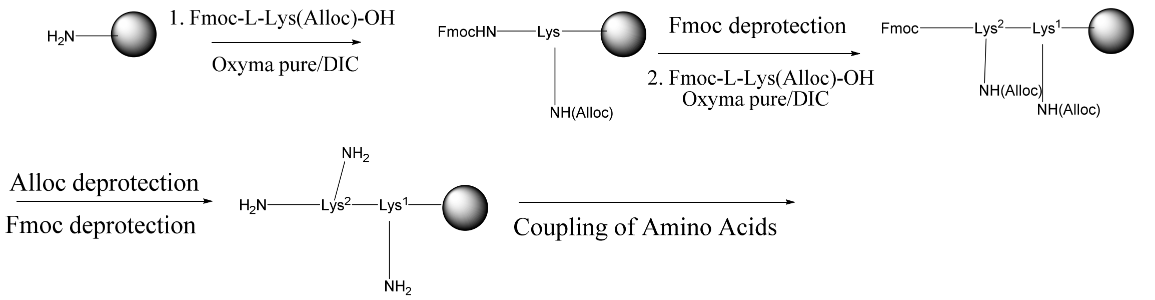

13, 26, 39 Fmoc-Phe-OH/Oxyma pure/DIC
Deprotection
12, 25,38 Fmoc-Leu-OH/Oxyma pure/DIC
Deprotection
11, 24, 37 Fmoc-Lys(Alloc)-OH/Oxyma pure/DIC
Deprotection
10, 23, 36 Fmoc-ala-OH/Oxyma pure/DIC
Deprotection
9, 22, 35 Fmoc-Leu-OH/Oxuma pure/DIC
Deprotection
8, 21. 34 Fmoc-Met-OH/Oxyma pure/DIC
Deprotection
7, 20, 33 Fmoc-Gly-OH/Oxyma pure/DIC
Deprotection
6, 19, 32 Fmoc-Val-OH/Oxyma pure/DIC
Deprotection
5, 18, 31 Fmoc-Ile-OH/Oxyma pure/DIC
Deprotection
4, 17, 30 Fmoc-Gly-OH/Oxyma pure/DIC
Deprotection
3, 16, 29 Fmoc-Ser-OH/Oxyma pure/DIC
Deprotection
2, 15, 28 Fmoc-Leu-OH/Oxyma pure/DIC
Deprotection
1, 14, 27 Fmoc-Phe-OH/Oxyma pure/DIC
Fmoc Deprotection
Cleavage
95% TFA, 1% TIPS, 2% H_2O, 2% Phenol

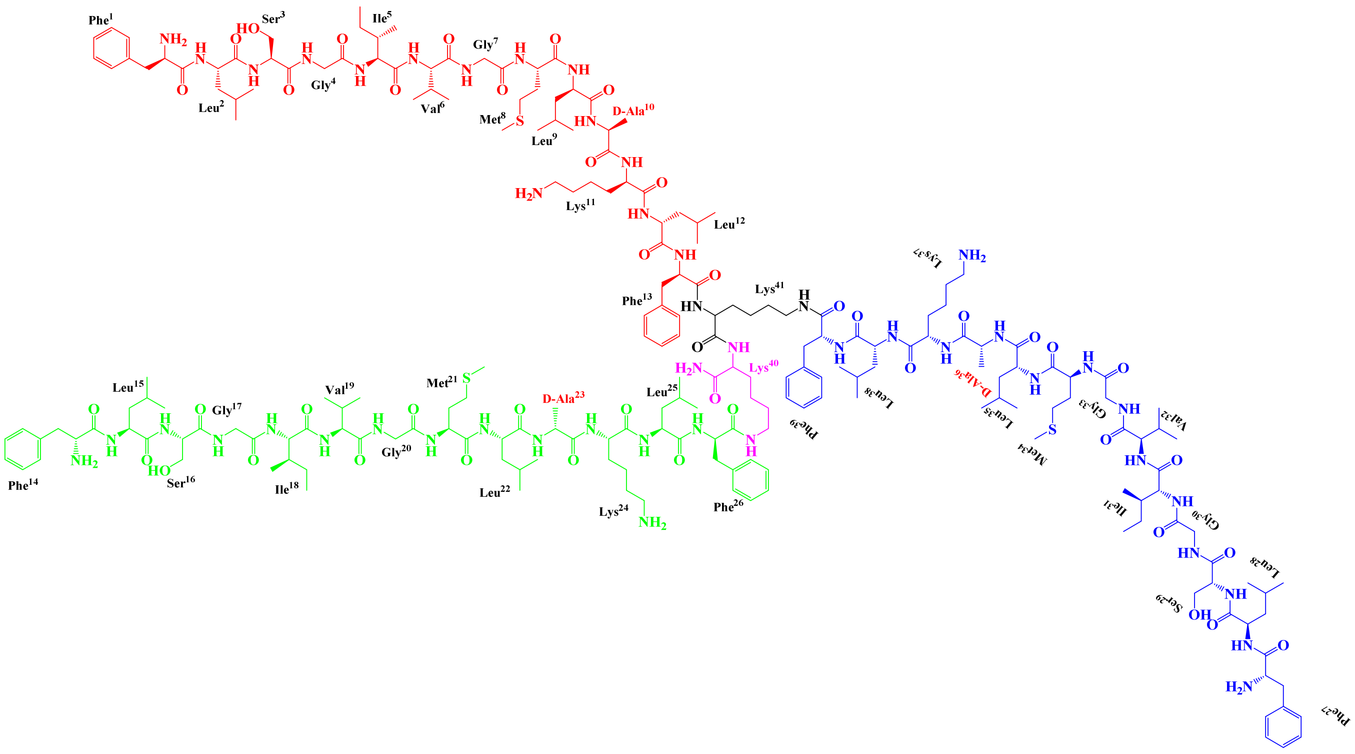

Figure S24. Synthetic scheme of [G10a]3-SHa

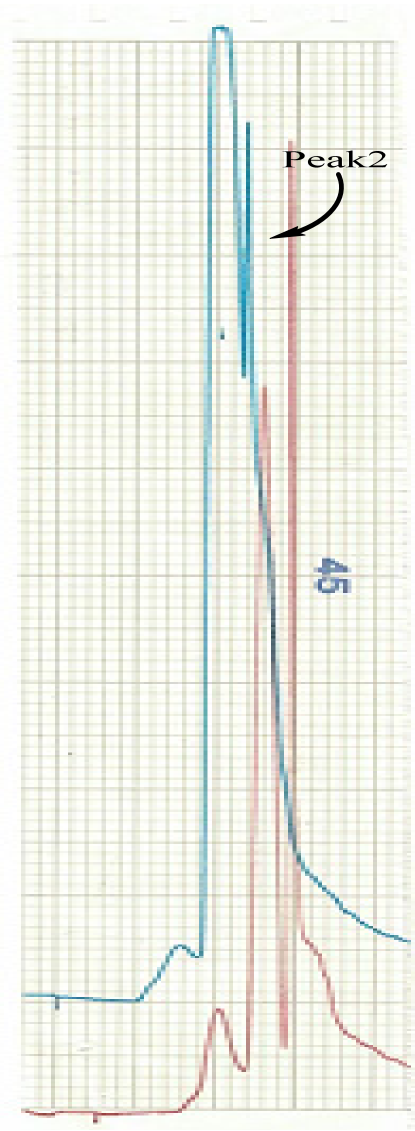

Figure S25: HPLC profile of [G10a]$_3$-SHa

2L-2 – Low Resolution ESI-MS

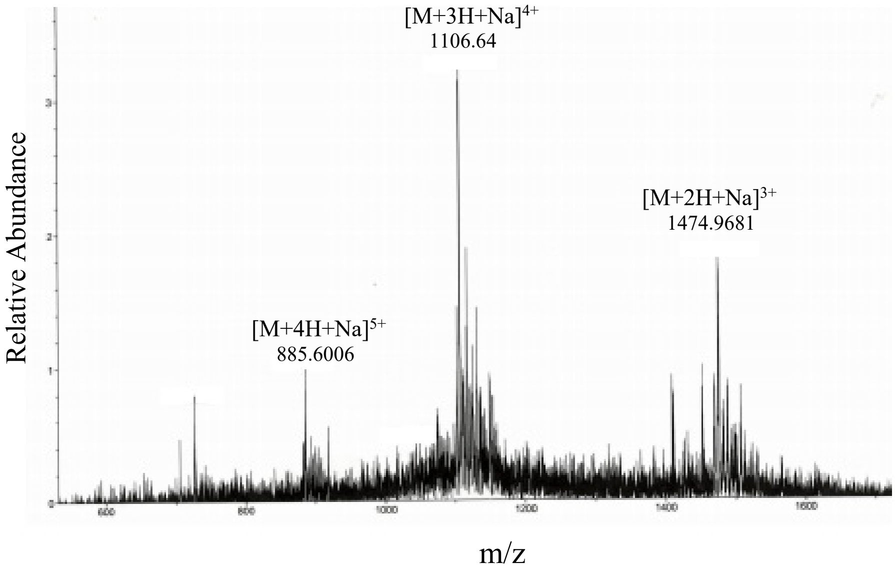

Figure S26: ESIMS (Low Resolution Positive Mode) of [G10a]$_3$-SHa

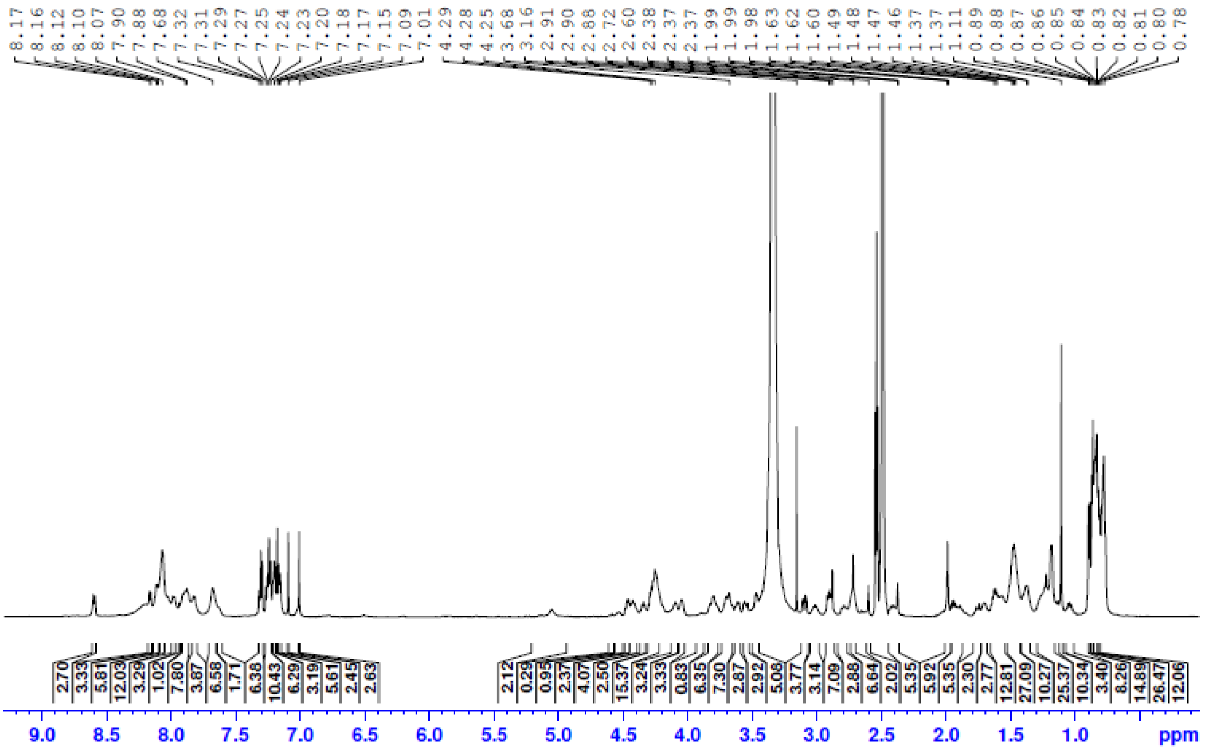

Figure S27: ^{1}H-NMR of [G10a]3-SHa

UV and IR analysis

[G10a]-SHa: White amorphous solid, UV (ACN) λmax (log ε) 218 (1.75); IR (KBr, cm−1) 3777.0 (NH), 3490.0 (OH), 3080.0 (=C-H), 2960.8 (C-H), 1670.1 (-NHC=O stretching), 1531.8 (Aromatic -C=C-, CN stretching, NH bending), 1443.7 (C-H bending, Aromatic -C=C- stretching), and 1200.7 and 1136.7 (C-O stretching, CN stretching, NH bending).

[G10a]$_2$-SHa: White amorphous solid, UV (ACN) λmax (log ε) 231 (1.176) nm; IR (KBr, cm−1) 3664.3 (NH), 3301.3 (OH), 3087.0 (=C-H), 2960.5 (C-H), 1666.8 (-NHC=O stretching), 1535.7 (Aromatic -C=C-, CN stretching, NH bending), 1438.6 (C-H bending, Aromatic -C=C- stretching), and 1202.7 (C-O stretching, CN stretching, NH bending).

[G10a]$_3$-SHa: Purple amorphous solid, UV (ACN) λmax (log ε) 220 (0.427) nm; IR (KBr, cm−1) 3662.4 (NH), 3296.4 (OH), 3074.0 (=C-H), 2962.1 and 2927.8 (C-H), 1668.6 and 1636.8 (-NHC=O stretching), 1533.9 (Aromatic -C=C-, CN stretching, NH bending), 1438.7 (C-H bending, Aromatic -C=C- stretching), and 1201.2 and 1135.2 (C-O stretching, CN stretching, NH bending).

Jeff-[G10a]$_2$-SHa: Pale yellow amorphous solid, UV (ACN) λmax (log ε) 229 (0.987) nm; IR (KBr, cm−1) 3661.7 (NH), 3295.8 (OH), 3084.7 (=C-H), 2928.4 (C-H), 1672.6 and 1635.1 (-NHC=O stretching), 1538.8 (Aromatic -C=C-, CN stretching, NH bending), 1437.8 and 1400.6 (C-H bending, Aromatic -C=C- stretching), and 1202.8 (C-O stretching, CN stretching, NH bending).

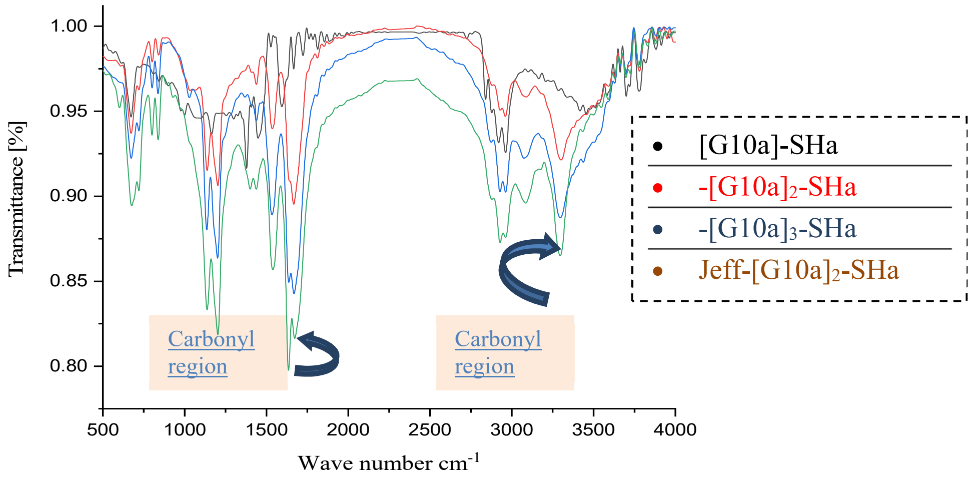

Figure S28: IR spectra of dendrimer peptides using KBr disc technique

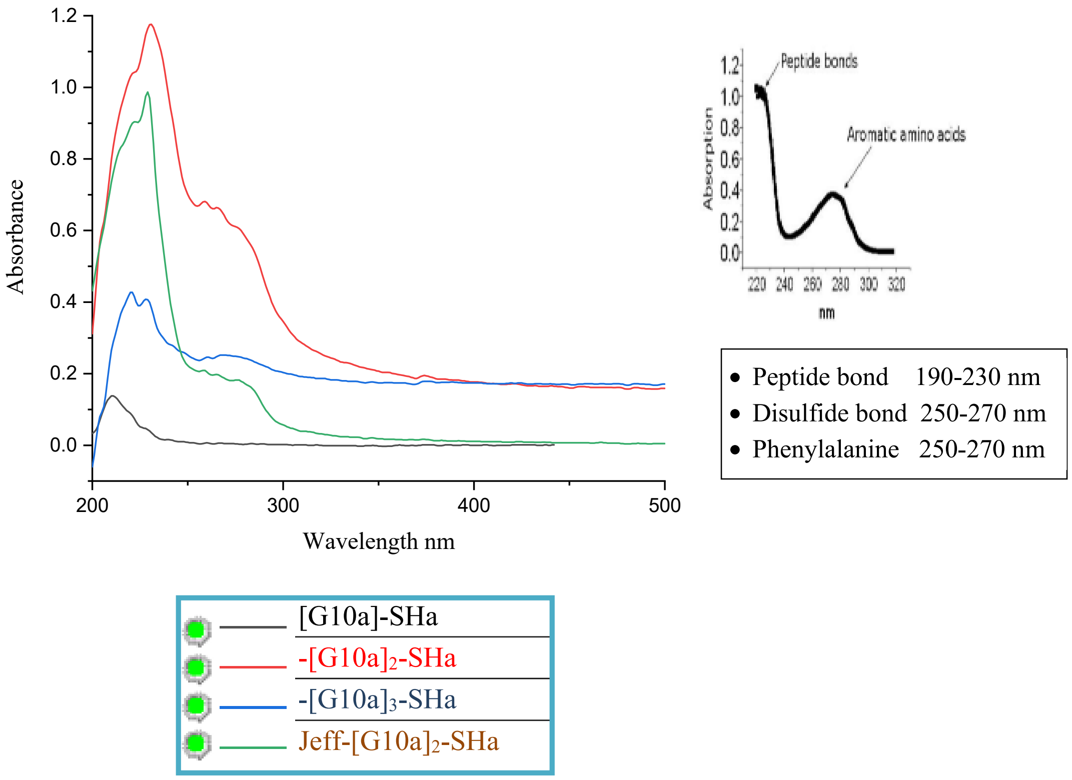

Figure S29: UV-Visible spectra of dendrimer peptides showing peptide bond and disulfide bond region
